# Revisiting the Immunological Landscape of Locoregional Therapies for Gastrointestinal Cancers: A Shift Toward Interventional Immuno-Oncology

**DOI:** 10.1007/s11912-026-01807-1

**Published:** 2026-07-03

**Authors:** Augustin Suffisseau, Johanne Seguin, Katia Lemdani, Sarah Diakhaby, Claude Capron, Mostafa El Hajjam, Fabrice Ruiz, Frederique Peschaud, Nathalie Mignet, Robert Malafosse

**Affiliations:** 1 Université Paris Cité, CNRS, INSERM, UTCBS, 4 Avenue de l’Observatoire, Paris, 75006 France; 2 Department of Digestive and Oncologic Surgery, Assistance Publique-Hôpitaux de Paris (AP-HP), Ambroise Pare Hospital, Université Paris-Saclay, 9 Avenue Charles de Gaulle, Boulogne-Billancourt, 92104 France; 3 Department of Interventional Radiology, Assistance Publique—Hôpitaux de Paris (AP-HP), Ambroise Pare Hospital, Université Paris-Saclay, 9 Avenue Charles de Gaulle, Boulogne-Billancourt, 92104 France; 4 Immunology Department, Assistance Publique-Hôpitaux de Paris (AP-HP), Ambroise-Pare Hospital, Université Paris-Saclay, 9 Avenue Charles de Gaulle, Boulogne-Billancourt, 92104 France; 5 EA4340 BCOH, Paris-Saclay University, Gif sur Yvette, 91190 France; 6 IMISCA Therapeutics, 110 Avenue Pierre Brossolette, Malakoff, 92240 France; 7 ClinSearh, 110 Avenue Pierre Brossolette, Malakoff, 92240 France

**Keywords:** Gastrointestinal cancers, Locoregional ablative treatment, Intratumoural immunotherapy, Tumour microenvironment, Interventional immuno-oncology

## Abstract

**Purpose of Review:**

Locoregional therapies have emerged as key components in the therapeutic arsenal against gastrointestinal cancers, offering minimally invasive options with curative potential. Beyond their direct cytotoxic effects, these interventions can remodel the tumour microenvironment by inducing immunogenic cell death and initiating local and systemic immune responses, including abscopal effects. This review aims to examine how these immunomodulatory properties may support locoregional therapies as an ideal platform for intratumoural immunotherapy, positioning their combination at the core of the evolving field of interventional immuno-oncology.

**Recent Findings:**

Recent studies demonstrate that locoregional therapies can induce immune responses through modulation of the tumour microenvironment, creating conditions that may be exploited by intratumoural immunotherapy. This review provides a comprehensive overview of current locoregional modalities, including thermal and non-thermal ablative techniques, transarterial therapies, and minimally invasive radiation methods, detailing their mechanisms of action, clinical applications, and immune-related effects. Special attention is given to emerging evidence supporting the combination of these approaches with intratumoural delivery of immunomodulatory agents, to enhance anticancer immune responses.

**Summary:**

The convergence of locoregional intervention and targeted immunomodulation represents an emerging therapeutic concept in the treatment of gastrointestinal cancers, offering the prospect of personalised, tumour-directed immune activation. This approach holds the potential to extend the benefits of immunotherapy beyond genetically defined subgroups to a broader patient population.

## Introduction

Gastrointestinal cancers account for a quarter of all new cancer cases, with nearly 5 million new cases each year. They are one of the leading causes of malignancy worldwide [1]. A large majority of patients will develop metastases during the course of their disease, leading to death for most of them. For patients with non-metastatic or oligo-metastatic disease, curative treatment is proposed with good long-term oncological outcomes. Surgical resection is the main curative strategy, but the location and size of the tumour may limit its feasibility. Chemotherapy and radiotherapy are combined for advanced stages. Immunotherapies have also been used as other therapeutic strategies, but only a small subset of patients (10%) with gastrointestinal malignancies with DNA mismatch repair deficiency (dMMR) or microsatellite instability (MSI-H) may benefit [2].

Locoregional ablative treatments are expanding the range of curative strategies available for advanced gastrointestinal cancers. These treatments offer less invasive curative techniques and can also be used as neoadjuvant treatments in addition to surgery in therapeutic management. Recent studies have focused on locoregional treatments and their potential to induce a systemic anti-tumour response through multiple pathways. In addition to physically destroying the tumour, this response may reduce the risk of local recurrence and contribute to the destruction of micrometastases through an abscopal effect [3].

The remodelling of the tumour microenvironment induced by locoregional ablative treatments opens up new perspectives for personalised approaches to cancer therapy. By generating tumour antigen release, inflammatory signalling, and local immune-cell recruitment, these treatments may create favourable conditions for targeted immunomodulation. Rather than relying solely on systemic immunotherapy in genetically defined subgroups of patients, local immunomodulatory strategies aim to amplify an anti-tumour response within a specific tumour microenvironment. Moreover, the immune response could lead to cytotoxic T cell response both locally at the tumour level and distally at potential metastases. This effect is known as the abscopal effect and has been described extensively [3]. In addition, the adaptive immune response against the tumour could lead to a lymphocyte memory and consequently the combination of anticancer therapy could be seen as a form of in situ tumour vaccination [4].

In this review, we will discuss the locoregional therapies used in clinical practice for the treatment of gastrointestinal cancers and their immunomodulatory effects. We will classify the therapies into three categories: physical ablation methods (distinguishing between thermal and non-thermal methods), transarterial therapies and minimally invasive radiation therapies. For each therapy, we will describe the technique, discuss the clinical application, and highlight the induced immunomodulatory effects. Finally, we will explore the possibility of combining these locoregional treatments with local immunotherapy and the prospects for future therapeutic strategies.

## Physical Methods

### Thermal Ablation

#### Radiofrequency Ablation (RFA)

RFA is the most widely used technique for the local ablation of primitive and metastatic liver tumours. It is a minimally invasive technique in which a single needle electrode is usually inserted into the tumour core and a dissipated current is delivered, either during laparotomy, laparoscopy, percutaneously, or endoscopically. The extent of cellular damage depends on three factors: the amount of energy applied (currently between 375 and 500 kHz), the time of application, and the sensitivity of the tissue to thermal damage. Lethal hyperthermia (50–100 °C) applied close to the needle will induces tissue coagulation and tumour necrosis [5]. In the periphery, sub-lethal hyperthermia (< 50 °C) produces a combination of necrosis, apoptosis, or recovery, depending on exposure time [6]. The ability of RFA to achieve complete tumour eradication appears to be dependent on the tumour size and location [7]. Tumours over 3 cm in diameter and tumours adjacent to vessels of 3 mm or more significantly reduce the rate of complete tumour ablation [8].

##### Clinical Outcomes of RFA

For unresectable patients with hepatocellular carcinoma (HCC), RFA is the recommended locoregional therapy [9, 10]. To date, randomised trials [11–15] or retrospective studies [16–19] comparing RFA with surgical resection have shown comparable efficacy for small HCC less than 3 cm in diameter, with fewer postoperative complications. However, outcomes remain strongly influenced by tumour size, location or liver function, and surgical resection remains preferred when technically feasible in suitable candidates [20].

In colorectal cancer (CRC), more than half of all patients will develop liver metastases during the course of their disease, and 25% of these patients will have liver metastases at the time of the diagnosis [21]. Although surgical resection remains the standard of care [22], nearly 80% of patients are not eligible due to hepatic tumour distribution, hepatic functional reserve, or the presence of extrahepatic disease. Many studies have demonstrated the outcomes of RFA for colorectal liver metastases; patients who may benefit should have liver metastases 3 cm or less in diameter, limited in number (< 5), distant from blood vessels or biliary ducts, no extrahepatic disease, or controllable lung metastases [23–25]. It can also be safely integrated into combined colorectal and liver procedures in selected cases [26]. Recently, the COLLISION trial demonstrated the non-inferiority RFA compared with surgical resection for eligible metastases, with similar overall survival and local control, and fewer adverse events [27]. In patients with unresectable disease, local ablation with RFA has also been associated with improved overall survival [28].

Apart from HCC and colorectal liver metastases, the clinical evidence supporting RFA is more limited and less practice-defining. RFA may represent a minimally invasive option for selected pulmonary metastases from CRC in non-surgical candidates, with reported progression-free survival rates of 35% at 2 years and overall survival of 69% at 3 years in retrospective series [29]. For gastric cancer liver metastases, indications remain unclear [30] and probably restricted to highly selected patients with metachronous and oligo-metastatic disease [31, 32] (NCT03042169). Retrospective studies suggest that RFA is feasible in patients who are not candidates for surgical resection and may provide better outcomes than chemotherapy alone, but robust comparative evidence is lacking [33, 34]. In Barrett-associated early oesophageal neoplasia, RFA is an effective method for complete eradication [35–37]. However, its use in early oesophageal cancer cannot be recommended in clinical practice in the absence of randomised trials [38]. In locally advanced pancreatic adenocarcinoma (LAPC), RFA remains investigational but have demonstrated safety and improved overall patient survival, with heterogeneous benefits between series [39, 40]. In addition, in recent work, authors have performed endoscopic ultrasound-guided RFA (EUS-RFA) with safety mainly for pancreatic neuroendocrine tumours [41–43].

##### Immunomodulation After RFA

**Fig. 1:**
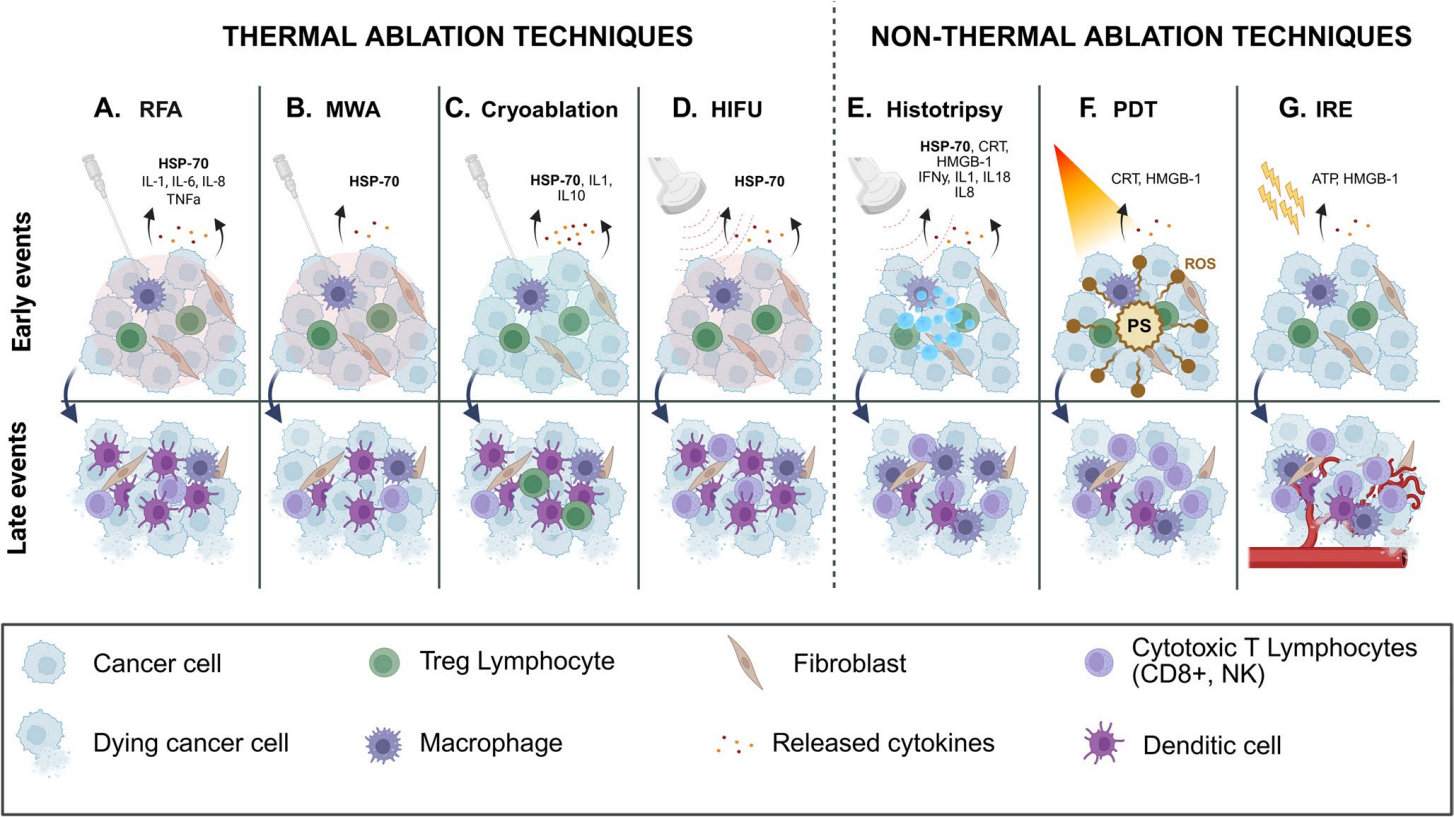
Immunological changes in the tumour microenvironment following tumour ablation. Thermal ablation techniques (**A**-**D**) and non-thermal techniques (**E**-**G**) are presented. For each method, the top of the figure illustrates how it works and the main molecules secreted following cell death. The bottom of the figure illustrates the immunological changes induced within the tumour microenvironment. Figure made using Biorender.com (Agreement number: NX292PVQV2)

Immunomodulation after RFA results from immune cell death (ICD) induced by the thermal effect. Coagulative necrosis occurs in the tumour core exposed to high temperature, resulting in protein denaturation and immediate release of antigens, extracellular matrix and DAMPs such as IL-1, IL-6, IL-8, TNF-α and HSP-70 [6, 44–46] Heat Shock Protein (HSP)−70, mainly released in the peripheral ablation area, is elevated in the serum after RFA, leading to maturation of dendritic cells (DC) and activation of a specific immunological anti-tumour response [47, 48]. (Fig. 1 A). Moreover, local modulation of the tumour microenvironment can enhance systemic antitumour immunity through the Abscopal effect, whereby immune responses initiated at the treated site spread to distant, untreated lesions [49]. However, in this peripheral zone, IL-6, HSP-70, and hypoxia-related pathways have also been reported to stimulate tumour outgrowth, potentially contributing to early recurrences [50–53].

Clinical evidence for RFA-induced immune responses is limited. Several data have shown that RFA can activate a specific immune response to the tumour antigen in patients with metastatic liver disease, associated with an increase in circulating T cells after stimulation with autologous tumour antigens [54, 55]. However, in patients with colorectal cancer who underwent liver resection several weeks after RFA, histological examination of lesions distant from the resected tumours showed no change in tumour infiltrating lymphocytes (TILs) levels [49].

In locally advanced pancreatic cancer (LAPC), Giardano et al. analysed pre- and post-operative peripheral blood from 10 LAPC patients and found that RFA activated the adaptative immune response by increasing CD4 + and CD8 + T-cell levels from day 3 and myeloid DCs levels at day 30, while maintaining the levels of Treg and other chemokines as TGF-β and TNF-α [56].

#### Microwave Ablation (MWA)

MWA is a thermal ablation technique used to treat liver tumours. MWA can generate very high temperatures in a very short period of time, ranging from 80 to 150 °C, which increases the effectiveness of this treatment on larger tumours [57]. Currently available MWA systems operate at frequencies of 915 MHz or 2.45 GHz. These wavelengths are less affected by changes in tissue impedance and can travel more efficiently through fibrous materials such as pancreatic tissue [58]. Multiple microwave electrodes can be used simultaneously to treat large or multiple tumours in the same procedure. This technology is an alternative to RFA in the treatment of liver metastases. However, when compared to other thermal technics, MWA ablation zones can be more difficult to delimit, can lead to overtreatment and may cause unintended thermal damage to adjacent structures.

##### Clinical Outcomes of MWA

MWA has been suggested as a way of overcoming some of the limitations of RFA, particularly the heat-sink effect and the difficulty of treating larger lesions measuring over 2.5 cm [59, 60]. Like RFA, MWA is predominantly used to treat HCC and colorectal liver metastases. In a case series of 219 patients with voluminous HCC (median tumour size of 3.2 cm), complete ablation could have been achieved in 97% of patients and the local recurrence rate was 8.5% at 11 months [61]. Recently, MWA has been considered an effective and safe way to treat early stage HCC adjacent to major vessels [62]. However, comparative data between MWA and RFA remain conflicting. A retrospective study comparing RFA and MWA for tumours larger than 2.5 cm showed no significant difference in local recurrence (21% versus 11%, *P* = 0.12) [63]. Moreover, a phase II trial, found that MWA was not more effective than RFA in treating HCC lesions of 4 cm or less, and the rate of local recurrence was similar for both techniques [64]. Wicks et al. recently showed no significant differences in overall survival in a large meta-analysis study of 31,000 patients comparing surgical resection, RFA, and MWA [65]. These results should be viewed with caution as MWA is a newer technique, and its increased use may change these data in future studies.

There is a paucity of data describing long-term outcomes with MWA for colorectal liver metastases. Eng et al. showed in a retrospective study of 33 patients that MWA is safe and feasible for the treatment of tumours smaller than 5 cm [66]. Other studies have reported comparable outcomes between MWA and surgical resection [67], or lower treated-site recurrence after MWA compared with RFA [68]. But the lack of recent prospective studies on the real clinical advantage of MWA compared to other local techniques prevents us from making a conclusion.

Outside HCC and colorectal liver metastases, the evidence supporting MWA remains more limited. MWA may represent a safe and useful option for selected pulmonary metastases [69], with reported major complication rates below 15%, no procedure-related mortality, 3-year local recurrence-free survival above 80%, and median overall survival of 58.6 months, depending on lesion size, ablation margin, and pleural involvement [70, 71]. For liver metastases from gastric cancer, the role of ablation remains debated. Small comparative and retrospective studies suggest that MWA may be feasible and may provide better outcomes than chemotherapy alone in selected patients with limited hepatic disease, depending on lesion size, number, and absence of extrahepatic disease [72, 73].

##### Immunomodulation After MWA

MWA induces an immune response similar to that described for RFA, with an upregulation of serum HSP-70 levels. (Fig. 1B). The extent of the immune response seems to be less with MWA [74]. Zhang et al. investigated the immune response induced by MWA [75]. They analyzed T cells (CD3, CD4, CD8), Treg, and NK cells in patients’ peripheral blood mononuclear cells (PBMC) one month after receiving MWA treatment. They found that T cell antitumour immunity is promoted by inhibition of Th2 cytokines, accompanied by increased Th1 cytokines, thus by local inflammatory injury and repair. However, there is a lack of data on this topic in clinical practice. Leuchte et al. demonstrated that, while MWA had only a moderate impact on circulating immune cell subsets, the stimulation of immune cells by major tumour-associated antigens enhanced tumour-specific immune responses in 30% of patients with hepatocellular carcinoma (HCC). Patients who exhibited this anti-tumour immune response showed longer progression-free survival (27.5 versus 10.0 months) [76]. To date, we have not found any relevant clinical data evaluating MWA-induced immune responses in colorectal carcinoma or pancreatic cancer.

#### Cryoablation

Cryoablation is a thermal ablation technique that uses the expansion of liquified gasses as nitrogen or argon from one or several cryoprobe implanted in the target lesion, through a mechanism known as the Joule-Thomson effect [77]. Cryoablation delivers temperatures around − 190 °C, and causes local tissue freezing, resulting in a combination of necrosis and delayed apoptotic cell death. The extreme cold also induces blood coagulation, resulting in vascular ischaemia of the tumour. The performance of cryoablation depends on four factors: the rate of cooling, the minimum temperature, the exposure time at this temperature during the procedure and the rate of thawing. Several freeze-thaw cycles are performed to achieve a complete ablation, with real-time monitoring to avoid injury to adjacent structures. The lack of coagulation effects on injured vessels can lead to haemorrhagic complications, unlike techniques that use temperature elevation.

##### Clinical Outcomes of Cryoablation

This newer method has a relatively limited use in clinical practice compared with RFA and MWA for the treatment of liver tumours. For HCC, Wang et al. compared RFA with cryoablation in a randomised controlled trial of 360 patients with tumours less than 4 cm in diameter. Similar overall and tumour-free survival rates were reported, but lower local tumour progression was shown with cryoablation than with RFA. (7.7% versus 18.2%, *P* = 0.041) [78]. Another large propensity-matched population study compared the two techniques and this time showed an advantage in liver cancer-specific survival for RFA [79]. However, in a smaller series of HCC in high-risk locations near major vessels, cryoablation appeared to be safer than MWA [80]. For both HCC and CRC metastases, Littrup et al. confirmed that percutaneous hepatic cryotherapy is safe with low local recurrence rates, lower than 12% after 1.8 years of mean follow-up, even for tumours close to vasculature and larger than 3 cm in diameter [81].

In lung tumours, the alveolar structure limits freezing and interferes with lesion destruction. After multiple freezing cycles, fluid and haemorrhage replace the air and fill the alveolus, leading to an estimated 20-fold increase in thermal conductivity [82]. In lung CRC metastases, a small clinical trial showed a 3-year local progression-free and overall survival rates of 59%, and 59.6%, respectively, depending on tumour size [83]. The ECLIPSE clinical trial confirmed the feasibility with low morbidity and one-year local control of over 90% [84].

Few articles have been published on the use of cryoablation for locally advanced pancreatic cancer (LAPC) using either open [85, 86] or percutaneous techniques [87, 88]. As monotherapy, cryotherapy demonstrated a median overall survival of 12.6 months for LAPC [87]. Cryotherapy has also been combined with brachytherapy with a median overall survival of 16.2 months, but with 6% major complications [88]. However, pancreatic cryoablation remains investigational. The freezing effect of cryoablation can injure surrounding tissues, including the duodenum, bile duct, or blood vessels, leading to inflammation, fistulas, or bleeding. Its use should therefore be restricted to highly selected patients and experienced centres.

##### Immunomodulation After Cryoablation

Cryoablation, like other thermal ablation techniques, has an immunostimulatory effect, with the ability to release mostly non-denatured proteins and induce profound dendritic cell (DC) loading [47, 89]. (Fig. 1 C). Even in extreme cases, a severe inflammatory response with a cytokine storm called ‘Cryoshock’ can be observed after cryoablation of voluminous tumours [90]. However, cryoablation has also been described to promote both immunostimulatory and immunosuppressive effects [91]. This immunosuppressive effect is partly explained by the increased levels of IL-10, an immunosuppressive cytokine that promotes Treg differentiation [92, 93]. In addition, some parts of the ablation zone may fail to induce immune cell death, preventing the generation of a complete anti-tumour immune response [44]. Therefore, it has been suggested that the rate of tissue freezing should be an important determinant of the immune response [94]. The literature on the immune response following cryoablation is limited to preclinical studies. Mauda-Havakuk et al. compared the immune response to cryoablation and RFA in a mouse model of colon cancer [95]. They found that cryoablation induced the production of a wider range of cytokines compared to RFA, with both pro- and anti-inflammatory properties. Cryoablation did not affect the number of Treg cells and had a less pronounced effect on anti-tumour immunity in the tumour microenvironment. In pancreatic cancer, White et al. compared irreversible electroporation (IRE) with cryoablation in a rodent model. They have found no significant changes in immune cells with cryoablation, compared to a strong intra-tumoural infiltration of macrophages and CD3 + T cells with IRE [96].

#### High-Intensity Focused Ultrasound (HIFU)

HIFU is a thermal ablation technique that uses externally applied ultrasound energy to cause thermal necrosis [97]. At higher power levels, tissue is subjected to frictional forces that are converted into heat. The target area is rapidly exposed to temperatures above 56 °C for a few seconds, resulting in thermal necrosis and immediate cell death. The optimal ultrasound frequency depends on the indication. It represents a compromise between the targeted depth and the desired heating rate. Therefore, frequencies range from 0.5 MHz for deep treatments to 8 MHz for shallower applications.

##### Clinical Outcomes of HIFU

Clinical applications of HIFU in the gastrointestinal system are limited to pancreatic and liver tumours but its clinical use is still uncommon due to anatomical challenges and the length of the procedure. Several series have been published demonstrating the safety and feasibility in hepatocellular carcinoma (HCC) [98–100]. Kim et al. compared the use of HIFU and TACE (Transarterial chemoembolization) with TACE alone in small HCC and showed a disease control rate based on RECIST criteria in favour of the combination group (78% vs. 54%; *p* = 0.035) with an increase in median survival time (57 months in the TACE + HIFU group vs. 36 months in the TACE alone group; *p* = 0.048) [101]. Nevertheless, the place of HIFU in therapeutic strategies for HCC remains unclear and may be considered mainly as a bridging option for patients awaiting liver transplantation [102].

Outside HCC, evidence remains scarce and based on small studies. In gastric cancer with liver metastases, HIFU has been used in patients who were contraindicated for either hepatectomy or RFA. Zhou et al. included 40 patients in a prospective study comparing HIFU with best supportive care (BSC) or palliative chemotherapy (PC). They showed a significant improvement in overall survival (27.5 months for HIFU vs. 7 months for BSC vs. 11.5 months for PC), progression-free survival (16.5 months for HIFU vs. 2 months for BSC and vs. 5 months for PC), and with no grade 3 or higher adverse events in the HIFU group [103]. For hepatic metastatic colorectal cancer, Yang et al. conducted a phase I clinical trial in 13 patients. No grade 3 or higher adverse events were reported, and they found a 2-year progression-free survival of 16.7% with a median overall survival of 25 months [104]. HIFU has also been used as a palliative treatment for locally advanced pancreatic cancer. Recent studies have shown that HIFU is safe and feasible and can improve the quality of life for these patients [105]. HIFU can provide pain relief in over 80% of patients, while also controlling tumour growth and producing a favourable clinical response [106].

##### Immunomodulation After HIFU

Thermal ablation by HIFU can induce immune responses in tumours by releasing intracellular molecular chaperones such as heat shock protein 70 (HSP70), leading to an increase in antigen-presenting cells in the microenvironment [107–109]. Recognition of tumour antigens by dendritic cells activates a T-cell-specific immune response [110] and increases tumour-infiltrating lymphocytes (TILs), particularly cytotoxic T CD8 + and natural killer (NK) cells [111].(Fig. 1D). Significant decreases in vascular endothelial growth factor (VEGF), TGF-β1, and TGF-β2 have also been observed after HIFU, in addition to tumour destruction [112]. Clinical evidence of immune stimulation after HIFU treatment of pancreatic cancer was observed. Thus Tonguc et al. highlighted a significant increase in IL-6 production 20 h after ablative therapy, associated with higher inflammatory response markers such as leukocyte levels, C-reactive protein (CRP) and lactate dehydrogenase (LDH) [113].

### Non-Thermal Ablation

#### Histotripsy

Histotripsy is the first non-thermal, non-ionising ultrasound-based ablation technique. Unlike HIFU, which uses continuous or long bursts of high intensity with a high duty cycle ultrasound (ultrasound on-time/total treatment time > 10%) to heat tissue, histotripsy uses short bursts of ultrasound with a low duty cycle (< 1%) to minimise heating [114] The key mechanism involves generating acoustic cavitation microbubbles, which expand and then collapse. This results in the mechanical disruption of cells and the release of cellular debris [115, 116]. The limitations of thermal devices, such as the heat sink effect and lack of precise margins, are overcome by histotripsy’s ability to preserve tissue, allowing it to be used in applications that are not feasible with thermal techniques. In addition, different tissues have specific resistance thresholds to histotripsy-induced damage, resulting in a tissue-selective, dose-dependent ablation [117].

#### Clinical Outcomes of Histotripsy

Histotripsy is still an emerging technique, with clinical evidence currently limited to early feasibility data. A proof of concept was achieved in a porcine liver tumour model, demonstrating effective tumour destruction by histotripsy while preserving critical anatomical structures of healthy tissue [118]. Several preclinical studies have shown promising results in various indications, leading to the THERESA trial, the first-in-human study using histotripsy in liver tumours [119]. This phase I study enrolled 8 patients with either hepatocellular carcinoma, cholangiocarcinoma, or metastatic colorectal or breast cancer. Technical success was achieved in all patients, and no device-related adverse events were observed.

Recent preclinical studies have also investigated the feasibility of histotripsy for pancreatic tumours. Results from a subcutaneous mouse model of pancreatic cancer showed that histotripsy could effectively ablate tumours and stimulate immune pathways [120]. Gannon et al. reproduced the technique in a porcine model. Histotripsy was therefore applied to the healthy pancreas of 11 pigs using ultrasound guidance. A pilot study was performed on 3 pigs, which failed to visualise the pancreas correctly due to the presence of gas in the overlaying bowel, resulting in off-target bowel injuries. The repetition of the technique in 8 pigs fed a suitable diet was successful, with 2 pigs showing off-target damage [121]. There are currently no published data on the clinical use of histotripsy in pancreatic cancer, but a feasibility study will be started in the next few years (NCT06282809).

#### Immunomodulation After Histotripsy

It has been suggested that histotripsy may induce a strong immune response compared to HIFU, due to the absence of denatured antigenic protein, but the mechanisms are not yet fully understood. An in vitro study showed that mechanical stress induced by cavitation microbubbles promoted immunogenic cell death of human breast cancer cells via the TNF-induced necrosis signalling pathway. It also increased Damage-Associated Molecular Patterns (DAMPs) like CRT, HSP70 and HMGB-1, pro-inflammatory cytokines (IFN-γ, IL-1α, IL-1β, IL-18) and chemokines (IL-8), associated with macrophage activation [122]. (Fig. 1E) These results have been confirmed in vivo in several cancer models, as nicely reviewed by Ashar et al. [123]. In a subcutaneous mouse model of pancreatic cancer, histotripsy was associated with a peptide and DNA release that was comparable to other non-thermal ablative techniques, and higher than thermal ones. An increase in tumour infiltrating lymphocytes was observed in the 24 h after treatment and decreased two weeks later [120].

#### Phototherapies (PDT))

Phototherapies such as visible wavelength photodynamic therapy (PDT) are local techniques that use light-responsive nanoparticles activated by a wavelength-matched laser or light. Standard PDT relies on three main elements, a photosensitiser (PS), light, and molecular oxygen to induce cell death through oxidative damage [124]. Briefly, a non-toxic PS is injected into a tumour or systemically. When the PS reaches a maximum concentration in the tumour as compared to healthy tissue, appropriate wavelengths of light excite the PS. Photothermal and photosensiting agent absorb energy from incoming photons and undergo a transition from an electronic ground state to an excited state. The excited state energy is transferred, among other de-excitation processes, to either the tissue substrate or surrounding oxygen and produces reactive oxygen species (ROS), specifically superoxide anion radicals and reactive singlet oxygen molecules [125, 126]. These high levels of ROS cause photodamage to proteins, lipids, and other molecules in the photosensitised area, resulting in the death of tumour cells by apoptosis and necrosis [127]. The characteristics of the light source, the delivery device, the optical properties of the tissues, and the concentration of the PS play a crucial role in the penetration of light into the tissue. With the accelerated development of nanotechnologies, the use of nanoparticles can provide tumour-specific targeting and avoid off-target systemic effects [128].

#### Clinical Outcomes of PDT

PDT can be used to treat intraluminal and superficial gastrointestinal cancers. These ablative techniques are considered the standard of care for endoscopic treatment of Barrett’s esophagus. In a prospective study of 208 patients with high-grade dysplasia, PDT reduced the degree of dysplasia and decreased the number of cancers that occurred in the treated group as compared with the control group [129]. However, due to local complications and phototoxicity, PDT has largely been replaced by the use of RFA, cryoablation, or submucosal dissection technique for the treatment of Barrett’s oesophagus [130]. Similarly, PDT was formerly used in the palliative treatment of obstructive esophageal adenocarcinoma, but its usage has decreased [131]. Although there is no clear indication for oesophageal PDT today, second-generation devices allow PDT to be used for small superficial oesophageal squamous cell carcinoma that is difficult to remove by conventional endoscopic resection, or in patients with bleeding risk, previous resection, or fibrosis [132]. PDT has also been proposed as a salvage treatment for non-surgical, local failure after chemoradiotherapy for oesophageal cancer [133, 134].

In other gastrointestinal indications, the clinical role of PDT remains limited. PDT was approved in the 1990 s, mainly in Japan, for early gastric cancer in patients who couldn’t undergo conventional endoscopic resection due to submucosal invasion [135]. However, the development of endoscopic submucosal dissection has replaced PDT even for lesions with an ulcer scar of 3 cm or less [136] PDT was also first used for cholangiocarcinoma in 1998 [137], with an improvement in the quality-of-life index and median survival time. However, several case series have been published with heterogeneous data, making the survival results unreliable [138]. The use of PDT in pancreatic cancer has also been reported. Preclinical studies in animal model of pancreatic cancer showed effective tumour necrosis after phototherapy, but with damage to neighbouring organs [139–141]. Some phase I trials of PDT have been published following preclinical reports, showing significant necrosis of pancreatic cancer with acceptable morbidity [142, 143]. To reduce PDT-related morbidity, strategies based on short-acting photosensitisers such as verteporfin and endoscopic ultrasound-guided delivery have shown local tumour necrosis with acceptable morbidity, supporting further evaluation of approaches designed to limit systemic phototoxicity [143, 144].

#### Immunomodulation After PDT

The ability of PDT to induce immune cell death (ICD) depends on the type of photosensitiser and the localisation of their action. ICD is maximal when phototoxicity is centered on intracellular organelles such as the endoplasmic reticulum or lysosomes [145, 146]. The destruction of the cancer cells and the peritumoural extracellular matrix releases tumour antigens such as DAMPs and induces inflammation. This local inflammatory response stimulates tumour-specific cytotoxic T cells capable of destroying both residual local cancer cells and distant untreated tumour cells [147]. Tanaka et al. showed in a murine colon cancer model that PDT with G-chlorine as PS induced the release of calreticulin (CRT) and high-mobility group box 1 protein (HMGB1). (Fig. 1 F). Mice inoculated with PDT-treated CT26 cells were significantly protected after a subsequent challenge with live CT26 cells [148].

#### Irreversible Electroporation (IRE)

IRE is a term used to describe oncolysis technologies and devices that induce cell death by repeated application of short-duration, high-voltage electrical pulses (HVEPs) up to 3000 V to cause permeabilization and destabilization of cell membranes, resulting in necrotic and delayed apoptotic cell death [149]. The non-thermal technique has several advantages. Firstly, HVEPs preserve the collagen matrix and adjacent structures close to the tumour, allowing for cellular regeneration. Secondly, non-thermal cell death does not denature immune proteins and keeps tumour antigens fully exploitable by the immune system [150]. In addition, HVEPs are not dependent on thermal conductivity and are not hindered by the heat sink effect of adjacent vessels. In these aspects, the ablation zone is very predictable and easy to monitor using ultrasound or computed tomography.

#### Clinical Outcomes of IRE

First clinical studies suggest that IRE preserves the structural integrity of the vessels and allows reliable treatment of liver tumours close to vital organ structures [151]. Clinical trials have shown that IRE is safe and feasible for both HCC and colorectal metastases (mCRC), with complete ablation ranging from 66% to 100% [152–156]. The COLDFIRE-2 clinical trial evaluated IRE for unresectable colorectal liver metastases. One-year progression-free survival was 68% and median overall survival from the first IRE was 2.7 years [157]. However, in liver tumours, IRE appears to show better outcomes for the treatment of HCC than for colorectal metastases [158]. The development of IRE systems is currently being evaluated in HCC (NCT01078415) and more data are needed to find a relevant place in treatment strategies for HCC. For unresectable perihilar cholangiocarcinoma, some teams have conducted pilot studies and demonstrated safety and efficacy with a 1-year survival rate reaching 75% [159, 160].

For locally advanced pancreatic cancer (LAPC), irreversible electroporation (IRE) is commonly performed intraoperatively during open laparotomy. Intraoperative ultrasound (US) is used to optimise ablation success and minimise complications. Several studies have described survival benefits for patients undergoing IRE, with median overall survival after diagnosis extended to 28 months [161–163]. PANFIRE-2 is the largest prospective cohort of 40 LAPC and 10 locally recurrent patients who underwent CT-guided percutaneous IRE, in which a median overall survival of 7.9 months after treatment was reported [164]. The most commonly reported major complications following IRE procedures include pancreatitis, ileus, portal vein thrombosis, pancreatic or biliary leaks, and intra-abdominal or digestive bleeding [165]. These complication rates in the literature vary between studies of percutaneous IRE (0–40%) but are generally lower compared to open IRE procedures (8–53%) [166].

For upper oesophageal cancer, research mainly focused on device development and has only reached the preclinical stages. Experimental animal studies in pigs have shown promising results [167, 168].

#### Immunomodulation After IRE

Immunomodulation after IRE has been described as the most potent technique compared to other ablation techniques, particularly for efficient protein release and T-cell activation [89]. Zhao et al. showed in an orthotopic mouse model of pancreatic cancer that IRE induced intratumoural hypoxia by increasing microvessel density and vascular permeability, which is a key factor in microenvironmental immunomodulation [169] (Fig. 1G). In clinical trials, especially in pancreatic cancers, IRE-induced immune response was observed through elevation of CD4 and CD8 T-cells expressing PD-1 and decrease of Treg. There was a trend for these T-cell responses to be associated with longer overall survival [170, 171].

### Transarterial Therapies

#### Selective Internal Radiotherapy (SIRT)

SIRT is based on resin or glass matrix microspheres to which β-emitter such as Yttrium-90 (Y90) or Holium-166 (Ho166) are attached. Because of the lack of a reel embolisation effect, the term selective internal radiotherapy (SIRT) is preferred to trans-arterial radioembolization (TARE). The most commonly used, Y90, emits a maximum energy of 2.27 MeV with a mean level of 0.93 MeV, and the maximum range of emission in the tissue is 11 mm with a mean of 2.5 mm [195]. Other radioactive materials have been used, such as iodine-131 or rhenium-188 labelled with lipiodol, but these are much less common [196]. The Y90-microspheres are injected directly into the target via the hepatic arterial system, allowing a maximum tumour dose and minimising radiations to adjacent tissues. Two types of microspheres are currently available on the market: the glass made TheraSphere^®^ and the resin made SeriSpheres^®^. TheraSphere^®^ has a low embolic power but a higher activity while SeriSpheres^®^ is suitable for larger lesions due to its higher embolic power but requires slower injection and angiographic control. The two approaches have not shown differences in term of efficacy in clinical trials [197].

#### Clinical Outcomes of SIRT

SIRT is most commonly used to treat HCC at different stages of the disease. Some authors have described “radiation segmentectomy” to successfully treat HCC in its early stages [198]. These results have been confirmed by the LEGACY trial, a retrospective multicentre study that included 162 patients with HCC smaller than 8 cm [199]. With a follow-up of 29.9 months, the authors reported an objective response rate (ORR) of 88.3% (CI: 82.4–92.4) and an overall survival of 86.6% at 3 years [200]. SIRT may be considered as an option for BCLC (Barcelona Clinic Liver Cancer staging system)-A HCC when resection or percutaneous ablation is non indicated [10]. In more advanced HCC, however, the survival benefit of SIRT remains less clearly established. The French SARAH trial compared SIRT (using Y90) to systemic sorafenib in this indication in 459 patients [201]. This study was negative, showing no difference between the two groups in terms of overall survival (8 months for SIRT vs. 9.9 months; *p* = 0.18) or disease-free survival (4.1 months vs. 3,7 months; *p* = 0,256). SIRT has been compared with TACE in 2 prospective randomised trials with small populations, showing improved time to progression but inconsistent overall survival benefit [199, 202]. Overall, SIRT appears most relevant in selected HCC patients, particularly when ablation or resection is not feasible, but its role varies according to tumour stage and comparator treatment.

SIRT has also been evaluated in liver metastatic colorectal cancer. The combined FOXFIRE-SIRFLOX-FOXFIRE-Global analysis of 1.103 patients evaluated the efficacy and safety of SIRT with 90Y resin microspheres in combination with first-line FOLFOX chemotherapy versus FOLFOX alone in patients with liver-only or liver-dominant metastases [203]. Despite an improvement in objective response rate, this combined analysis was negative, showing that the addition of SIRT did not affect overall survival and increased the incidence of grade III-IV major adverse events. However, these prospective clinical trials suggest that liver surgery after SIRT is safe.

For intrahepatic cholangiocarcinoma, SIRT has been evaluated as a potential downstaging strategy in initially unresectable disease. Retrospective and phase II data suggest that Y90-based SIRT, alone or combined with chemotherapy, may facilitate secondary resection in selected patients, with reported R0 resection rates of around 20% in prospective data [204, 205]. However, these results remain preliminary and are insufficient to define SIRT as a standard indication in cholangiocarcinoma.

#### Immunomodulation After SIRT

**Fig. 2:**
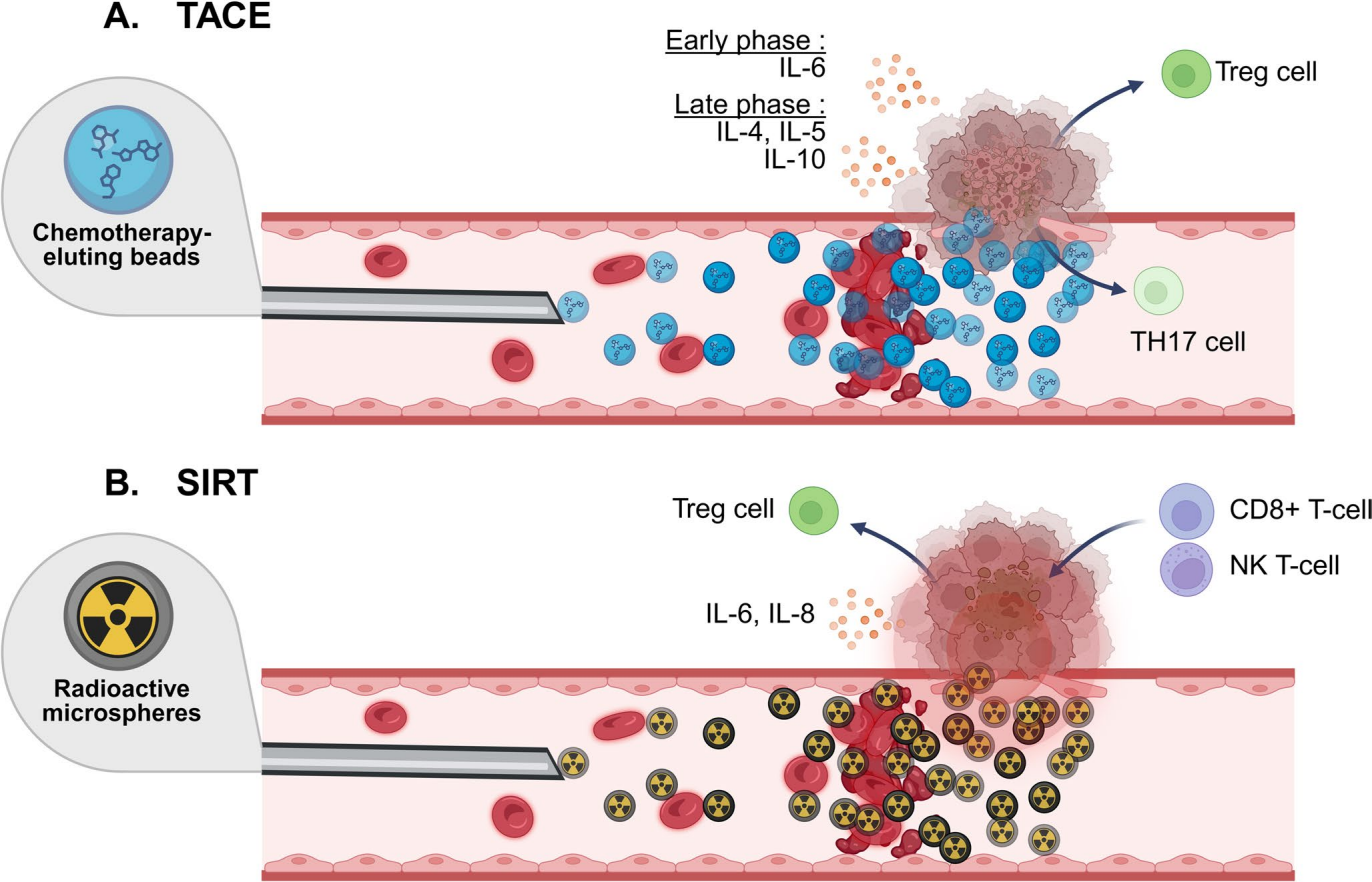
Visual representation of the technical modalities of arterial embolisation therapies, along with the locoregional immunomodulation induced by these treatments. (**A**) Transarterial chemoembolisation (TACE) (**B**) Selective internal radiotherapy (SIRT). Figure made using Biorender.com (Agreement number: MN292PVA21)

Selective radioembolisation with Y90 microspheres induces local and systemic immune responses. Cancer cell death increases the tumour antigen presentation, activates CD8 T-cells and NKT cells, and decreases the proportion of Treg in the TILs population. TARE also activates pro-inflammatory cytokines such as IL-6 and IL-8, whose baseline levels are used as biomarkers and are associated with liver function and survival in patients with HCC [206–208] (Fig. 2B). These immunostimulatory effects have allowed for safety studies combining radioembolisation and immunotherapy, particularly immune checkpoint inhibitors (ICI) [209, 210] for HCC.

### Transarterial Chemoembolization (TACE)

TACE is a minimally invasive technique in which chemotherapeutic drugs are delivered locally through a catheter placed in a targeted artery. Selective drug delivery adjacent healthy tissue to be spared by selective arterial occlusion. The concept of TACE relies on tumour arterial neoangiogenesis to deliver high concentrations of chemotherapy. For this reason, tumours with abundant blood vessels may benefit more from regional infusion, resulting in higher localised drug concentrations [172]. Cytotoxic drugs can be emulsified in Lipiodol, a radio-opaque contrast agent, like the conventional TACE technique (cTACE). The direct infusion of chemotherapeutics is followed by embolisation of the tumour-feeding artery with a piece of gelatin sponge. The other technique, called DEB-TACE, uses drug-eluting beads loaded with chemotherapy drugs. These non-resorbable beads are used to simultaneously deliver absorbed chemotherapy and embolize the feeding artery, resulting in hypoxia and necrosis of tumour cells [173]. The delivery of the chemotherapy is based on plasma ion exchange as a function of time, resulting in sustained concentrated local delivery of the chemotherapeutic agent. Increased concentrations, reduced toxicities, and potentially improved tumour response may occur in the hopes of altering or regulating tumour biology, resulting in an improved survival.

#### Clinical Outcomes of TACE

TACE is a standard of care for liver tumours, particularly HCC [174]. TACE is the first-line palliative treatment for patients with advanced HCC without metastases and in good general condition, with a 20% increase in overall survival rate at 2 years [175, 176]. This makes HCC the most established indication for TACE in gastrointestinal oncology.

In colorectal liver metastases, TACE can be used in 3 situations: as a neo-adjuvant therapy to achieve better downstaging of lesions and allow the surgery (NCT02885753) [177–180], as an adjuvant therapy after liver resection to reduce the recurrence rate [181], and as palliative therapy in chemoresistant disease to increase overall survival rate [21]. The phase 3 PACHA-01/PRODIGE 43 [182] (NCT02494973) and SULTAN UCGI30/PRODIGE 53 [183] (NCT03164655) trials should provide new elements to define the place of TACE in the strategies to treat colorectal liver metastases. Today, irinotecan-eluting beads (DEBIRI) are used to treat patients with metastatic colorectal cancer. Fiorentini et al. reported improved overall survival with DEBIRI compared with systemic FOLFIRI chemotherapy in a phase 3 study of 74 patients (22 vs. 15 months, *p* = 0.031) [184]. Other phase 2 studies suggested improved downsizing, but without consistent improvement in progression-free survival and without a clear reduction in adverse events compared with systemic chemotherapy [185, 186]. Overall, these data support the feasibility of DEBIRI-based strategies, but remain insufficient to firmly establish their role in colorectal liver metastases.

In localised pancreatic carcinoma, regional intra-arterial approaches (RIAC) have also been evaluated. The difference between RIAC and TACE lies in the absence of embolisation for RIAC, which is only a chemo-infusion in a targeted area. Liu et al. reported a meta-analysis of 6 randomised controlled trials comparing systemic chemotherapy with regional intra-arterial chemotherapy, showing increased median overall survival and fewer overall and haematological adverse events [187]. Several series have also evaluated TACE in liver metastatic pancreatic cancer, with median overall survival ranging up to 16–19 months [188, 189]. However, these data are mostly derived from selected series and heterogeneous intra-arterial strategies. Therefore, the role of TACE or related regional intra-arterial approaches in pancreatic cancer remains investigational and should not be considered established in routine practice.

#### Immunomodulation After TACE

TACE has been shown to increase the peripheral circulation of T helper cells mediated by Th17 cells. A decrease in Treg levels has also been observed in patients [190, 191]. TACE also induces an acute, early-phase increase of IL-6 levels at 3 days post-procedure, and a late-phase increase in the Th2 cytokines IL-4, IL-5, and IL-10 at 2 months post-procedure [192] (Fig. 2 A). Hiroishi et al. demonstrated a CD8 + T cell response against tumour antigens in patients with HCC. In this work, this T-cell response was the only prognostic factor for prolonged tumour-free interval (*p* = 0.022) [193]. In hepatocellular carcinoma, TACE has been shown to enhance intratumoural expression of PD-1 and PD-L1 on both tumour cells and infiltrating lymphocytes, reinforcing the concept of TACE as a loco-regional inducer of immunogenic cell death that simultaneously triggers immune checkpoint upregulation [194].

## Minimally Invasive Irradiation Therapies

### Stereotactic Body Radiotherapy (SBRT) and Stereotactic Ablative Radiotherapy (SABR)

Stereotactic body radiotherapy (SBRT) and stereotactic ablative radiotherapy (SABR) are minimally invasive technologies that can deliver a large amount of highly focused doses of ionizing radiation to the target tumour, sparing the surrounding normal tissue [211]. The term SABR should be used for stereotactic therapies delivered with curative intent to patients with oligo-metastatic disease, whereas the term SBRT should be used to describe radiation therapy delivered with palliative intent, regardless of overall disease burden. In addition to more precise imaging modalities, modern stereotactic techniques use gating and tracking models that have made focal treatment more efficient. Percutaneous platinum or gold fiducial markers, safely placed under US- or CT- guidance, in the core and margins of the liver lesions, appear to be useful for stereotactic strategies [212]. In the near future, MRI guidance systems may overcome fiducial marker implantation [213].

#### Clinical Outcomes of SBRT/SABR

Stereotactic radiotherapy has been developed over the past decade, primarily for the treatment of lung cancer, but is now increasingly being used for gastrointestinal cancers [214].

Liver tumours are a target of choice for SBRT. It has been recognized as an alternative therapy for HCC patients who are ineligible for standard ablation modalities or transplantation. SABR has been associated with improved 3-year local control and OS rates of approximatively 84% and 48%, respectively, and low grade 3 toxicity with no specific mortality [215]. A recent meta-analysis evaluated local control rates of 80%, and future research should determine the efficacy of SBRT compared to other local treatments [216]. For liver metastases from colorectal cancer, Gil-Raga et al. showed a median overall survival of 28.9 months (95% CI; 19–38.7) in 49 selected nonsurgical patients treated with a combination of SBRT and standard treatments, with minimal side effects and no patients with grade 3–4 adverse events [217]. Based on the current outcome data, SBRT or SABR are valuable tools in the therapeutic approach for patients with liver metastases and can be used as an alternative or adjunct to minimally invasive ablative procedures [218, 219]. However, the optimal place of SBRT in the treatment pathway, between surgery and thermal ablation or systemic therapy, remains to be determined.

SABR has also been evaluated in locally advanced pancreatic cancer. Kisivan et al. reported the feasibility of pancreatic SABR using peritumoural fiducials, with no grade > 2 adverse events [220]. Rossi et al. published a series of 52 selected locally advanced pancreatic carcinomas treated with SABR after completion of induction chemotherapy [221]. After a median follow-up of 16.1 months, the median overall survival and progression-free survival were 29.7 and 8.7 months, respectively. Surgery was feasible in 26.6% of patients and was associated with improved survival. Although these results support feasibility in selected patients, the oncological benefit of SABR in pancreatic cancer remains uncertain and requires prospective evaluation.

#### Immunomodulation After SBRT/SABR

**Fig. 3:**
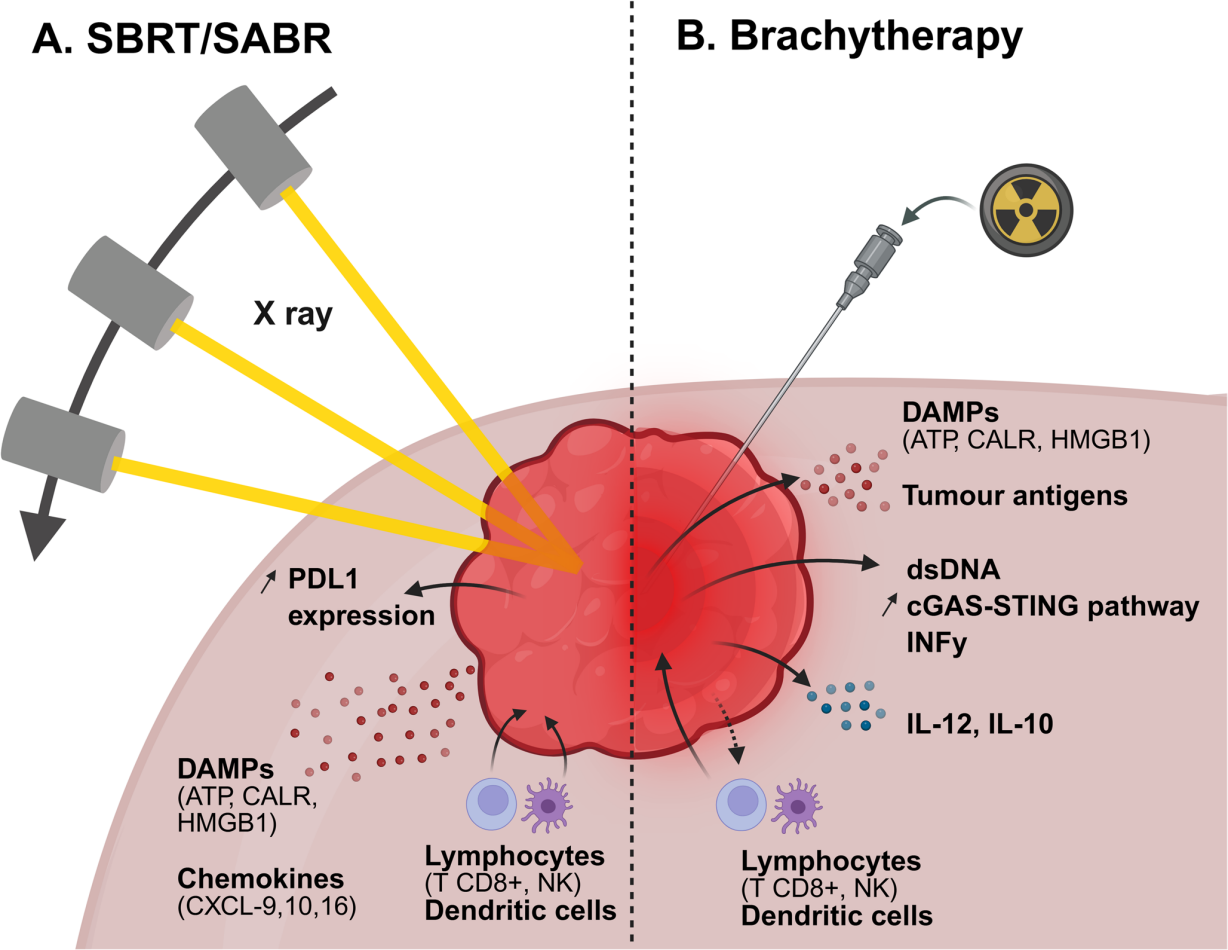
Modification of the tumour microenvironment induced by minimally invasive radiation therapies. This figure illustrates changes in intra-tumour immune cell populations, as well as immunomodulatory chemokines secreted systemically. **A**. Stereotactic body radiotherapy (SBRT) and stereotactic ablative radiotherapy (SABR) **B**. Brachytherapy. Figure made using biorender.com (Agreement number: SI292PVI8O)

In addition to locoregional ablative effects, locally delivered radiotherapy can induce a systemic anti-tumour immune response that is even more potent than conventional radiotherapy [222]. The immune effect of standard radiotherapy begins with DNA modification induced by radiation waves, leading to cell death and the release of DAMPs. These factors include the release of high mobility group box 1 (HMGB1) nuclear protein by dying tumour cells, which interacts with Toll-like receptor 4 (TLR4) on dendritic cells (DCs) [223]. Radiotherapy can also promotes the recruitment of cytotoxic CD8 T cells to the tumour microenvironment through the release of chemokines such as CXCL9, CXCL10 and CXCL16, the induction of intracellular adhesion molecule (ICAM)−1 and vascular cell adhesion molecule (VCAM)−1, and the upregulation of MHC class 1 molecules or NKG2D receptor stress ligands on the surface of tumour cells [224]. (Fig. 3 A) However, well-documented cases of abscopal effect with radiotherapy alone are sporadic in the clinic. In contrast to the positive immunostimulatory effects, radiotherapy may promote the generation of immunosuppressive mediators such as tumour-associated macrophages (TAMs), myeloid-derived cells (MDSC) and PDL-1 expression, which downgrade the activation of DCs and impair the function of effector T cells [225]. SABR seems to be an answer to this dual effect. Indeed, SABR induces a more robust immune response by releasing a large amount of tumour-associated antigens and enhancing CD8 + T cell infiltration. SABR is also able to spare the radiation dose to the draining lymph nodes, allowing T cell priming [226]. A second perspective is the combination of specific immunotherapeutic agents with radiotherapy. SABR appears as a sustainable choice for this combination thanks to its high and durable local control, giving a favourable window for the effect of immunotherapies [227].

### Brachytherapy

Brachytherapy consists in placing radioactive sources within or very close to the tumour tissue. A high dose of radiation can then be delivered to the tumour while limiting harmful effects on normal tissue. Since the discovery of radium in the 19th century and its use in skin cancer, several radioactive sources have been used because of their specific physical properties. These properties will determine the dose rate (high > 12 Gy/h; low between 0.4 and 2 Gy/h; very low < 0.4 Gy/h), but also the rhythm and duration of brachytherapy. The technique also depends on the delivery of the radioisotope, and modern image guidance systems (US- CT- or MRI-), which brachytherapy safer and more adaptable [228]. The use of brachytherapy is now widespread and has a place in the treatment of several gastrointestinal cancers [229].

#### Clinical Outcomes of Brachytherapy

In gastrointestinal oncology, brachytherapy has been evaluated across several tumour sites, but its level of clinical evidence varies substantially according to indication. In oesophageal cancer, high-dose rate brachytherapy with 192Ir has been used both as definitive treatment for unresectable tumours and for palliative purposes. However, despite its inclusion in selected indications according to the American Brachytherapy Society guidelines, early studies reported substantial toxicity, including treatment-related mortality and fistula, reaching 10% and 12% respectively [230, 231]. In palliative situations, endoluminal brachytherapy is well documented and allows local control of more than 10 months in irradiated area [232]. In cholangiocarcinoma, intraluminal brachytherapy using 192Ir source combined with biliary stenting has suggested improved outcomes in obstructive biliary malignancies where surgery is not feasible, but evidence remains based on small prospective and retrospective studies [233–236]. Similarly, percutaneous interstitial high-dose-rate brachytherapy has demonstrated feasibility and local control in selected liver malignancies, including colorectal metastases and HCC, particularly for lesions unsuitable for thermal ablation because of size or proximity to vessels [237–244].

In pancreatic ductal adenocarcinoma (PDAC), Iodine-125 seed implantation has been used for palliative purposes by open surgery [245–248] or percutaneously under CT- or US- guidance [88, 249–251]. Reported median overall survival ranges from 7.3 to 11 months for percutaneous local therapy alone, with complication rates varying from 0 to 17% [252]. More recently, EUS-guided phosphorus-32 (32P) implantation has shown encouraging feasibility in the PanCO pilot study [253], with a local control rate of 90.5% in the per-protocol analysis, and is currently being evaluated in the prospective randomized TRIPP-FFX trial (NCT05466799).

For non-metastatic anal squamous cell carcinoma, brachytherapy is used as a complementary boost after external beam radiotherapy [254]. Although the ACCORD 03 trial showed no benefit for a total radiation dose higher than 60 Gy [255], retrospective studies have suggested lower 5-year local recurrence rates with a brachytherapy boost compared with conventional radiotherapy [256, 257]. This supports a possible role for brachytherapy as a boost strategy in selected patients, but the level of evidence remains limited.

Contact X-ray brachytherapy has the strongest contemporary evidence among gastrointestinal brachytherapy indications, particularly for organ preservation in rectal cancer [258]. The aim is to deliver an internal radiotherapy beam through an intracavitary applicator, allowing a higher local radiation dose to be delivered with fewer adverse events. The Lyon R96-02 phase III trial demonstrated significantly higher sphincter preservation rates than standard radiotherapy, with a 10-year cumulative rate of permanent colostomy of 29% in the interventional group versus 63% in the control group [259]. The recent phase III OPERA trial enrolled patients with rectal adenocarcinoma who were treated with either standard chemoradiotherapy and an external beam boost of 9 Gy or a contact-X-ray brachytherapy boost. This trial confirmed this organ-preservation benefit, with a 3-year organ preservation rate of 81% in the contact-X-ray brachytherapy group versus 59% in the external beam boost group, and lower local regrowth at 5 years (17% versus 39%) [260, 261]. This supports the use of this technique as a treatment of choice for resectable locally advanced rectal cancer.

#### Immunomodulation After Brachytherapy

Brachytherapy, like the rest of the radiation therapies, induces an anti-tumour immune response mediated by the release of DAMPs and subsequent activation of the immune system (Fig. 3B). Intra-tumoural implantation of radioactive seeds allows a very high dose of radiation locally in the tumour and may also lead to necrosis. There is no specific literature on immunomodulation after brachytherapy, but advantages can be extrapolated from well-known mechanisms of external beam radiotherapy. First, normal tissues crossed by external beam radiotherapy are damaged and radiosensitive immune cells contained in these tissues may be depleted [262]. The long-term lymphopenia that can be induced may be limited by brachytherapy, which allows for poor off-target effects. Secondly, brachytherapy produces radiation heterogeneity, with high doses delivered close to the source and progressively lower doses delivered with distance from the source. Various immunogenic mechanisms are involved, each with different dose-response profiles [263]. The central high-dose region (> 12 Gy) is the optimal site for maximal cell death and release of tumour antigens. High-intermediate dose region (8–12 Gy) can induce release of dsDNA and cGAS/STING/interferon-γ which drive phenotypic changes in immune sensitivity on surviving tumour cells. Moderate dose region (2–5 Gy) may optimize the release of stimulatory cytokines, such as IL12 and IL10, and enhanced immune cell infiltration through the expression of ICAM-1 and VCAM. The low dose region (1–2 Gy) may induce a transient local depletion of infiltrating lymphocytes, which could create a window during which the tumour immune microenvironment could be restored in the absence of other suppressive mechanisms. Finally, brachytherapy offers the possibility of combining treatments and enhancing the effect of intratumoural therapies such as immunotherapies.

## Perspective of Combination with Local Immunotherapy

In gastrointestinal cancers, immunotherapy represents a true revolution. Systemic immunotherapies, especially Immunologic Checkpoint Inhibitors (ICI), have demonstrated their efficacy in inducing durable antitumour immune responses and in increasing overall survival in patients with DNA mismatch repair deficiency (dMMR) or microsatellite instability (MSI-H) [2]. The abnormality of DNA synthesis or repair generate a high mutational charge and high level in synthesis of abnormal proteins in cancer cells. These tumours have massive infiltration of cytotoxic T cells and high PD-L1 expression, resulting in response to ICI.

For the large majority of gastrointestinal cancers, genetically stable (MSS-H), immunotherapies are inefficient [264]. Reversing this resistance to immunotherapy is the key to success for future therapeutic strategies. Prognosis in these tumours is also strongly influenced by the tumour microenvironment, in particular the density and spatial organisation of tumour-infiltrating lymphocytes [265]. Many targets are beginning to be explored, and one way is to modify the tumour microenvironment, transforming a “cold tumour” into a “hot one” [266].

**Fig. 4:**
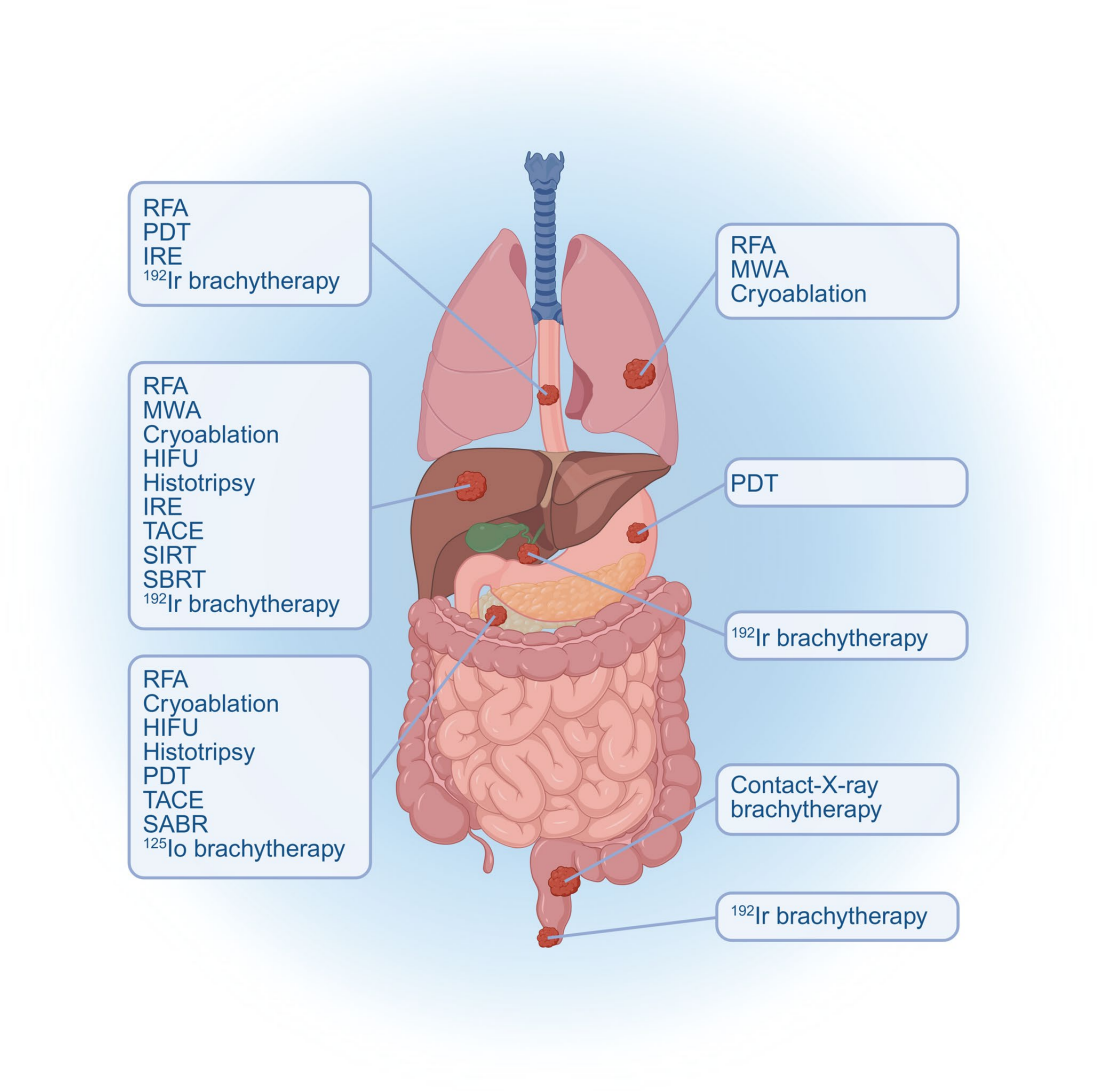
Overview of the various locoregional ablation techniques that can be used depending on the tumour’s location. Figure made using Biorender.com (Agreement number: DZ292PVUN2)

As we have shown in the previous part of this review, the use of locoregional ablative treatment in the antitumoural strategy provides these conditions for priming an efficient antitumoural immune response. This response includes tumour antigen release, inflammatory signalling, danger-associated molecular patterns release, and immune-cell recruitment., both locally in tumour micro-environment and systemically. The Fig. 4 summarizes the diversity of locoregional therapies and their target. This wide range of therapies enables the most technically feasible option to be selected for each organ and tumour site. The multiple immunomodulatory effects that we have highlighted in this review open a new field of antitumour strategies by considering the combination with immunotherapy adapted to the ablative technique.

However, the clinical maturity of this concept remains heterogeneous. Some locoregional therapies are already established for local tumour control or organ preservation in selected gastrointestinal cancers, whereas others are supported mainly by retrospective series, feasibility studies, or early-phase trials. Moreover, most clinical studies were designed to evaluate technical success, local control, survival, or toxicity, rather than immune activation or systemic antitumour effects. Nevertheless, the convergence of preclinical evidence, translational findings, and early clinical signals supports interventional immuno-oncology as a promising field of research. By bringing together these data, this review aims to highlight the rationale for combining locoregional therapies with immunomodulation, while underlining the need for prospective studies incorporating immune monitoring and clinically meaningful outcomes.

### Benefits of Local Immunotherapy Toward Systemic Immunotherapy

The combination of ablative approaches and immunotherapy for cancer treatment is a very dynamic field. Clinical trials and future perspectives have already been reviewed several times [267, 268]. Synergetic interaction between tumour ablation and systemic immunotherapy showed preliminary, but promising results for the future of cancer treatment. However, systemic immunotherapy may be limited by poor tissue penetration. In a European phase II study, combining local ablation with RFA and systemic durvalumab and tremelimumab did not improve survival compared with standard treatment for unresectable liver metastases from colorectal cancer [269]. Adverse pharmacokinetics and a wide off-target distribution in healthy tissues can also be responsible of several adverse events. Most patients experience side effects, including systemic inflammation and autoimmune damage, which may lead to treatment disruption.

Local immunotherapy may overcome most of the disadvantages of standard drug delivery and expands anti-cancer strategies. Firstly, the bioavailability of the drug to the tumour and its direct drainage system is superior, offering the possibility of administering lower doses with similar efficacy and a reduction in major systemic toxicity [270, 271]. In addition, the immunosuppressive tumour microenvironment protects against infiltration by cellular mediators of cancer immunosurveillance, leading to the ineffectiveness of systemic immunotherapies [272]. Intra-tumoural or loco-regional administration of immunotherapies overcomes this barrier and enhance local inflammation in the tumour. Preclinical studies have shown that local administration of immune stimulants alone can induce the release of cytokines and pattern recognition receptor (PRR) agonists, thereby restoring the antitumour effect of immune checkpoint inhibitors [273, 274]. These observations have led to several clinical trials in the past few years [275] and have been supported by the FDA approval of talimogene laherparepvec (T-VEC) for intratumoural oncolytic virotherapy for melanoma in 2015 [276].

### Combination of Local Ablation Techniques and Local Immunotherapy - Ongoing Trials and Future Direction

We reviewed preclinical (Table 1) and clinical (Table 2) trials published or in progress, in which the authors combined locoregional therapy with the intratumoural injection of immunotherapy to treat gastrointestinal cancers. Analysis of the preclinical studies summarised in Table 1 highlights several consistent trends in the current landscape of combined local ablation and intratumoural immunotherapy. Most models focus on hepatic malignancies, either hepatocellular carcinoma or colorectal liver metastases. This reflects both the technical feasibility of repeated percutaneous injections and their current accessibility in clinical practice. Nevertheless, a growing number of studies are now focusing on pancreatic ductal adenocarcinoma. Recent work by Narayanan et al. and Ledezma et al. illustrates the increasing interest in combining ablative strategies with local immunotherapy for this highly immunosuppressive tumour type [277, 278]. The development of interventional endoscopy could benefit future clinical trials for oesophageal or primitive colorectal cancer.

**Table 1:** Literature review of preclinical studies evaluating locoregional ablative therapy combined with local immunotherapy in the treatment of gastrointestinal cancers. The references are categorised by treatment modality and target tumour type. Abbreviations - *PRRa* pattern recognition receptor agonist, *DC* dendritic cell, *CRC* colorectal cancer. In these preclinical trials, CRC models are ectopic models, representing liver-metastatic disease. *HCC* hepatocellular carcinoma, *IT* intratumoural

Ablation technique	Gastrointestinal cancer	Category of immunotherapy	Agent	Species studied – Tumor cell line	Administration route	Year	Reference
RFA	CRC	Cytokine	huKS-IL2	Balb/c mice – CT26-KS cells	IT injection	2009	[288]
	CRC	Dendritic cell	OK-432-stimulated DCs	C57BL/6 mice – MC38 cells	IT injection	2014	[289]
	CRC	Cytokine + PRRa	GM-CSF + HKMT (TLR2a) GM-CSF + BCG (TLR4a) GM-CSF + PPD (TLR4a)	Balb/c mice – CT26-Luc cells	IT injection	2019–2024	[49, 279, 280]
	HCC	Antibody	(131)I-chTNT	NZ white rabbits – VX2 cells	IT injection	2014	[290]
	HCC	PRRa	CpG B (TLR9a)	NZ white rabbits – VX2 cells	IT injection	2016	[281]
MWA	HCC	Cytokine	GM-CSF microspheres	C57BL/6 mice – Hepa 1–6 cells	IT injection	2009	[291]
	HCC	Cytokine	IL-2 microspheres	C57BL/6 mice – Hepa 1–6 cells	IT injection	2018	[292]
Cryoablation	HCC	Oncolytic virus	Cowpea mosaic virus (CPMV)	C57BL/6 mice – RIL-175 cells	IT injection	2023	[293]
	CRC/Pancreas	Cytokine	IL-12	C57BL/6 mice – MC38 and Panc02 cells	IT injection	2023	[294]
	CRC	PRRa	Imiquimod (TLR7a)	Balb/c mice – CT26 cells	IT injection	2024	[282]
HIFU	CRC	Cytokine + PRRa	pFAR4-IL-12 + TLR2a	Balb/c mice – CT26-Luc cells	IT injection	2022	[283]
PDT	CRC	Dendritic cell	Naïve DCs	Balb/c mice – CT26 cells	IT injection	2006	[284]
IRE	HCC	PRRa	poly-ICLC (TL3a) c-di-GMP (STINGa)	C56BL/6 mice and NZ white rabbits – Hepa.129 and VX2 cells C56BL/6 mice - PM299L cells	IT injection	2019–2021	[285, 286]
	Pancreas	PRRa	1V270 (TLR7a)	C56BL/6 mice - KPC4580P cells	IT injection	2019	[277]
	Pancreas	Oncolytic virus	Alphavirus M1	C57BL/6 mice –Panc02 cells	IT injection	2021	[295]
	Pancreas	Antibody Oncolytic virus	CD40Ab Cowpea mosaic virus (CPMV)	B6/129 F1J mice - KPC46 organoids	IT injection	2023–2025	[278, 296]
TACE	HCC	PRRa	CpG B (TLR9a)	Sprague Dawley rats – N1S1 cells	Embolisation	2023	[297]
SBRT	Pancreas	Antibody	CD40Ab	C57BL/6 mice – Panc02 cells	IT injection	2018	[298]
	CRC	Dendritic cell	Naïve DCs	Balb/c mice – CT26 cells	IT injection	2019	[299]

**Table 2:** Literature review of achieve or in progress clinical trials evaluating locoregional ablative therapy combined with local immunotherapy in the treatment of gastrointestinal cancers. The references are categorised by treatment modality and target tumour type. Abbreviations - *PRRa* pattern recognition receptor agonist, *DC* dendritic cell, *CRC-LM* liver metastases from colorectal cancer, *HCC* hepatocellular carcinoma, *IT* intratumoural

Ablation technique	Type of tumor	Category of local immunotherapy	Agent	Systemic co-therapy	Trial Status	Phases	Reference
RFA	HCC	Autologous cells	OK-432-stimulated DCs	-	Published	I/II	[287]
	CRC-LM	Synthetic immune stimulant	IP-001 (1% N-dihydro-galacto-chitosan)	-	Recruiting	I/II	NCT05688280 (INJECTABL-1)
	CRC-LM	Autologous cells	Autologous DCs	-	Terminated (lack of funding)	I	NCT00185874
MWA	Liver cancer, lung cancer	Oligodéoxynucléotides	CpG-ODN	CART cells	Recruiting	I	NCT04952272
IRE	Pancreas	PRRa	IMO-2125 (TLR9 agonist)	Nivolumab	Recruiting	I	NCT04612530
	Pancreas	Antibody	Mitazalimab (CD40 antibody)	-	Recruiting	I	NCT06205849
SBRT	Pancreas	Cytokine	PCX12	-	Not yet recruiting	I	NCT06217666
	CRC-LM	Antibody	Ipilimumab (Anti CTLA4)		Phase I terminated	I/II	NCT01769222
	CRC-LM	PRRa	Vidutolimod (TLR9 agonist)	Ipilimumab, Nivolumab	Completed	I	NCT03507699
Standard radiotherapy	Solid tumours	Cytokine	RiMO-401 (Indoleamine 2,3-dioxygenase 1 (IDO1) inhibitor)	-	Recruiting	I	NCT06182579
	HCC	Dendritic cell	Autologous DC	Pneumococcal 13-valent Conjugate Vaccine, Atezolizumab, Bevacizumab	Recruiting	I/II	NCT03942328

Across the variety of ablative approaches represented, thermal techniques, particularly RFA and MWA, remain the most widely used platforms for combination strategies, as illustrated by the work of Lemdani et al. and Seguin et al. in colorectal cancer metastatic models [49, 279, 280]. However, irreversible electroporation emerges as another dynamic area of research, with an expanding range of immunotherapeutic agents evaluated in hepatic and pancreatic models. In particular, Ledezma et al. recently combined IRE with intratumoural cowpea mosaic virus nanoparticles in an orthotopic metastatic PDAC murine model. They demonstrated improved survival, with an increased CD8⁺ T-cell and NK-cell infiltration at both primary and metastatic sites, as well as enhanced dendritic cell maturation and effector-memory CD8⁺ T-cell expansion. This is consistent with the induction of a systemic antitumour immune response [278].

Regarding immunotherapeutic agents, a striking feature in Table 1 is the predominance of dendritic cell–centred and PRR agonists strategies. In colorectal cancer models, Lemdani et al. and Seguin et al. combined RFA with intratumoural GM-CSF, bacterial-derived TLR agonists in a hydrogel-based formulations, achieving robust local and distant tumour control and providing a proof of concept for local immunomodulation in synergy with RFA [49, 280]. Similar approaches involving TLR3, TLR7, TLR9 and STING agonists in liver and pancreatic models support the notion that dendritic cells and their maturation via PRR signalling play a pivotal role in local immune modulation [277, 281–286]. Taken together, these preclinical studies provide a coherent mechanistic rationale for combining ablation-induced antigen release with local immune stimulation. However, they remain heterogeneous in terms of tumour models, ablative techniques, immunotherapeutic agents, timing or endpoints.

In the clinical setting, the trials summarised in Table 2 reveal similar trends, but at a earlier stage of maturity. Most studies have focused on hepatic tumours, including hepatocellular carcinoma and colorectal liver metastases, as well as pancreatic ductal adenocarcinoma. This reflects both the predominance of these diseases in interventional oncology and the practical feasibility of image-guided intratumoural administration. Another recurrent feature is the use of systemic immune checkpoint inhibitors, most frequently nivolumab and ipilimumab, in addition to local immunotherapy. This strategy aims to enhance the adaptive immune response triggered by local stimulation, particularly through the upregulation of PD-L1 following local ablative treatments. However, the clinical evidence remains dominated by early-phase trials, many of which are ongoing and primarily designed to assess safety, feasibility, dose escalation, or immune correlates rather than definitive oncological benefit. The most mature published clinical data remain limited to small phase I/II study, such as intratumoural dendritic cell injection after RFA for HCC [287]. In this 30-patient study, percutaneous injection of OK432-stimulated monocyte-derived dendritic cells after RFA was safe, with no grade 3–4 adverse events and was associated with longer recurrence-free survival compared with basic-protocol dendritic cells (24.8 vs. 13.0 months; *P* = 0.003). Interestingly, patients with increased tumour-associated antigen-specific T-cell responses had higher 5-year recurrence-free survival, supporting the biological relevance of local immune activation in this setting.

Overall, Tables 1 and 2 illustrate the central message of this review: interventional immuno-oncology is supported by a rich and increasingly consistent preclinical background, together with growing early clinical interest, but remains at the beginning of its clinical validation in gastrointestinal cancers. The available data provide a strong rationale for further development of combinations tailored to tumour site, immune contexture and technical accessibility. However, these approaches should currently be considered promising investigational strategies rather than validated treatments for routine clinical practice.

## Conclusion

The multiple immunomodulatory effects induced by local therapies provide a rationale for adapting treatment strategies to tumour location, immune contexture, and technical feasibility, rather than relying exclusively on systemic approaches. The field of local immunotherapy is expanding rapidly and encompasses a wide variety of immunomodulatory agents, including microorganisms such as oncolytic viruses or bacteria, as well as synthetic compounds such as PRR agonists, monoclonal antibodies, cytokines, nucleic acids and chimeric proteins. This diversity enables tailored interventions to target distinct steps in the cancer–immunity cycle. Furthermore, combining agents with complementary mechanisms can enhance antitumour effects by acting on multiple immune pathways simultaneously and potentially promoting more durable clinical responses [275].

However, the main limitation of intra-tumoural or locoregional immunotherapies remains the physical inaccessibility of many tumour sites. Overcoming this challenge will require clinical innovation and strong industry partnerships to accelerate the development of delivery systems that maximise local bioavailability and sustain immune activation at the tumour site. Advances in interventional radiology and therapeutic endoscopy may progressively enable the targeting of previously inaccessible tumour sites through precise, image-guided administration. Meanwhile, the refinement of pharmaceutical carriers including lipid-, nano-, and vesicle-based vectors, as well as hydrogel formulations, opens the way for tailored biodistribution profiles that concentrate immunotherapeutic agents within the tumour and its draining lymphatic network [279, 300–304]. The convergence of technological, pharmaceutical and interventional expertise is defining the emerging field of interventional immuno-oncology, a paradigm in which local immune modulation is integrated into minimally invasive procedures and has the potential to expand therapeutic options for patients with gastrointestinal cancers.

More data from pre-clinical experiments and clinical trials are needed to optimize combinatory treatment strategies. In particular, future studies will need to clarify patient selection, optimal timing, choice of immunomodulatory agents, delivery systems, immune monitoring, and clinically meaningful endpoints. Fully exploiting the potential of interventional immuno-oncology will require redefining partnerships between researchers, pharmacists, oncologists, surgeons, endoscopists, interventional radiologists and industrials. Close, patient-centred collaboration will support the development of more personalised and adaptable therapeutic pathways Thus, interventional immuno-oncology should currently be viewed as a promising but still developing area of personalised cancer treatment, aiming to induce tumour-specific immune responses, while requiring further translational and clinical validation.

## Key References

- Galon J, Bruni D. Approaches to treat immune hot, altered and cold tumours with combination immunotherapies. Nat Rev Drug Discov. 2019;18:197–218. 10.1038/s41573-018-0007-y*(Of importance)*.
  - ○ This review sets out a conceptual framework for combination immunotherapies designed to overcome tumour immune resistance.
- Kepp O, Marabelle A, Zitvogel L, Kroemer G. Oncolysis without viruses — inducing systemic anticancer immune responses with local therapies. Nat Rev Clin Oncol. 2020;17:49–64. 10.1038/s41571-019-0272-7*(Of importance)*.
  - ○ This review summarizes how local tumour destruction can induce systemic anticancer immune responses and support combination immunotherapy strategies.
- Seguin J, El Hajjam M, Legagneux J, Diakhaby S, Mignet N, Boudy V, et al. Radiofrequency Combined with Intratumoral Immunotherapy: Preclinical Results and Safety in Metastatic Colorectal Carcinoma. Pharmaceutics. 2024;16:315. 10.3390/pharmaceutics16030315*(Of outstanding importance)*.
  - ○ This preclinical study shows promising results of combining radiofrequency ablation with intratumoural immunotherapy in metastatic colorectal cancer models, with additional safety data in a large animal model.
- Ledezma DK, Joshi U, Nguyen-Ta K, Sonowal H, Russo H, Newton IG, et al. Irreversible electroporation with intratumoral plant virus immunotherapy induces systemic immunity in a metastatic model of pancreatic cancer. Cancer Lett. 2025;634:218074. 10.1016/j.canlet.2025.218074*(Of outstanding importance)*.
  - ○ This recent preclinical study demonstrates that irreversible electroporation combined with intratumoural immunotherapy induces systemic antitumour immunity and improves survival in a metastatic pancreatic cancer model.
- Van Der Lei S, Puijk RS, Dijkstra M, Schulz HH, Vos DJW, De Vries JJJ, et al. Thermal ablation versus surgical resection of small-size colorectal liver metastases (COLLISION): an international, randomised, controlled, phase 3 non-inferiority trial. Lancet Oncol. 2025;26:187–99. 10.1016/S1470-2045(24)00660-0*(Of outstanding importance)*.
  - ○ This major phase III trial establishes the non-inferiority of thermal ablation compared with surgical resection for small colorectal liver metastases, supporting its broader clinical use.
- Ruarus AH, Vroomen LGPH, Geboers B, van Veldhuisen E, Puijk RS, Nieuwenhuizen S, et al. Percutaneous Irreversible Electroporation in Locally Advanced and Recurrent Pancreatic Cancer (PANFIRE-2): A Multicenter, Prospective, Single-Arm, Phase II Study. Radiology. 2020;294:212–20. 10.1148/radiol.2019191109 (Of importance).
  - ○ To date, this study is the largest trial evaluating the feasibility and safety of percutaneous irreversible electroporation in locally advanced and recurrent pancreatic cancer.
- Baron D, Pace Loscos T, Schiappa R, Barbet N, Dost E, Ben Dhia S, et al. A phase III randomised trial on the addition of a contact X-ray brachytherapy boost to standard neoadjuvant chemo-radiotherapy for organ preservation in early rectal adenocarcinoma: 5 year results of the OPERA trial. Ann Oncol Off J Eur Soc Med Oncol. 2025;36:208–15. 10.1016/j.annonc.2024.10.827*(Of importance)*.
  - ○ To date, this is the largest study of contact-X-ray brachytherapy for treating early rectal adenocarcinoma, achieving major results in terms of organ preservation.
- Kitahara M, Mizukoshi E, Terashima T, Nakagawa H, Horii R, Iida N, et al. Safety and Long-Term Outcome of Intratumoral Injection of OK432-Stimulated Dendritic Cells for Hepatocellular Carcinomas After Radiofrequency Ablation. Transl Oncol. 2020;13:100777. 10.1016/j.tranon.2020.100777*(Of importance)*.
  - ○ This study is the first clinical trial to evaluate the feasibility of combining RFA and intratumoural immunotherapy for treating hepatocellular carcinoma.

## Data Availability

Not applicable.
